# A High Copy Suppressor Screen for Autophagy Defects in *Saccharomyces arl1*Δ and *ypt6*Δ Strains

**DOI:** 10.1534/g3.116.035998

**Published:** 2016-12-12

**Authors:** Shu Yang, Anne Rosenwald

**Affiliations:** Department of Biology, Georgetown University, Washington, DC 20057

**Keywords:** *Saccharomyces cerevisiae*, *ARL1*, *YPT6*, autophagy, rapamycin

## Abstract

In *Saccharomyces cerevisiae*, Arl1 and Ypt6, two small GTP-binding proteins that regulate membrane traffic in the secretory and endocytic pathways, are also necessary for autophagy. To gain information about potential partners of Arl1 and Ypt6 specifically in autophagy, we carried out a high copy number suppressor screen to identify genes that when overexpressed suppress the rapamycin sensitivity phenotype of *arl1*Δ and *ypt6*Δ strains at 37°. From the screen results, we selected *COG4*, *SNX4*, *TAX4*, *IVY1*, *PEP3*, *SLT2*, and *ATG5*, either membrane traffic or autophagy regulators, to further test whether they can suppress the specific autophagy defects of *arl1*Δ and *ypt6*Δ strains. As a result, we identified *COG4*, *SNX4*, and *TAX4* to be specific suppressors for the *arl1*Δ strain, and *IVY1* and *ATG5* for the *ypt6*Δ strain. Through this screen, we were able to confirm several membrane traffic and autophagy regulators that have novel relationships with Arl1 and Ypt6 during autophagy.

Autophagy is a conserved process for engulfing cytosolic components (proteins, organelles, etc.) into a double-membraned structure, the autophagosome, which subsequently fuses with the lysosome (the vacuole in *Saccharomyces cerevisiae*) (Nair and Klionsky 2005; Glick *et al.* 2010). Autophagy provides building blocks during starvation and removes damaged or unnecessary organelles. Thus, autophagy is indispensable for intracellular homeostasis (Yang and Klionsky 2010). Autophagy can be divided into selective and nonselective types, depending on the substrates. The nonselective form of autophagy, called macroautophagy, is responsible for the turnover of cytosolic components. In this paper, “autophagy” will be used to refer to macroautophagy.

Autophagy is complex and highly regulated. The master regulator is the Target of Rapamycin complex 1 (TORC1), a serine/threonine kinase that functions to sense the cell’s energy status. When cells have sufficient nutrients, TORC1 phosphorylates several substrates and blocks autophagy. In contrast, when cells are starved or treated with rapamycin, the TORC1 kinase is inhibited, triggering autophagy. The elongation of a structure called the phagophore to form the autophagosome requires Atg8, with a covalently-linked molecule of phosphatidylethanolamine (PE) on its C-terminus, which serves to recruit membranes to the phagophore (Klionsky *et al.* 2007).

Autophagy requires several specific proteins, the Atg proteins, but also requires transport of membrane vesicles to the phagophore assembly site (PAS). In yeast, many small GTP-binding proteins, membrane traffic regulators, are essential for different stages of autophagy, from the formation of the autophagosome to the fusion of the autophagosome and the vacuole (Yang and Rosenwald 2014). We recently discovered that two small GTP-binding proteins, Arl1, a member of the Arf/Arl/Sar family, and Ypt6, a member of the Rab family, have novel roles in autophagy in *S. cerevisiae* (Yang and Rosenwald 2016). Both regulate membrane traffic between the *trans*-Golgi network (TGN) and endosomes. *ARL1* and *YPT6* also show synthetic lethality with one another, suggesting the encoded proteins have functional similarities (Costanzo *et al.* 2010). Mutants lacking one or the other of these genes have similar phenotypes; both the *arl1*Δ and *ypt6*Δ strains are unable to grow in the presence of rapamycin and perform autophagy, but only at high temperature (37°). We determined that the high temperature defect is caused by a failure to recruit the Golgi-associated retrograde protein (GARP) complex to the PAS in the absence of *arl1*Δ or *ypt6*Δ at 37°. The GARP complex is responsible for recruiting the SNARE Tlg2 to the PAS to deliver membranes derived from the Golgi apparatus to the growing phagophore (Reggiori *et al.* 2003).

Here, we utilized a high copy genomic library (Nasmyth and Reed 1980) to perform a suppressor screen on *arl1*Δ and *ypt6*Δ strains. Since we found that neither deletion mutant strain can grow in the presence of rapamycin at 37°, we first used this assay to identify suppressors of the growth defects. Because we are interested in membrane traffic or autophagy regulators that have relationships with Arl1 or Ypt6 during autophagy, a total of seven genes (*COG4*, *SNX4*, *TAX4*, *IVY1*, *PEP3*, *SLT2*, and *ATG5*) were selected from the sequenced genomic fragments and were transformed into both the *arl1*Δ and *ypt6*Δ strains. Autophagy-specific assays were used to determine which genes suppressed the autophagy defects. As a result, we have identified novel partners of Arl1 or Ypt6 during autophagy.

## Materials and Methods

### Yeast strains, plasmids, and reagents

Yeast strains *arl1Δ* (*MATa/α his3Δ1/his3Δ1 leu2Δ0/leu2Δ0 LYS2/lys2Δ0 met15Δ0/MET15ura3Δ0/ura3Δ0, arl1Δ::KanMX/arl1Δ::KanMX*) and *ypt6Δ* (*MATa/α his3Δ1/his3Δ1 leu2Δ0/leu2Δ0 LYS2/lys2Δ0 met15Δ0/MET15ura3Δ0/ura3Δ0, ypt6Δ::KanMX/ypt6Δ::KanMX*) were used in the high copy suppressor screen for rapamycin sensitivity at 37°. Both strains were obtained from the homozygous diploid deletion collection developed by the *Saccharomyces* Genome Deletion Project (Winzeler *et al.* 1999); the parental strain is BY4743. Strains YSA003 (*pho8::pho8Δ60 arl1Δ::HIS3*) and YSA004 (*pho8::pho8Δ60 ypt6Δ::HIS3*) in the BY4742 background (*MATα his3Δ1 leu2Δ0 lys2Δ0 ura3Δ0*) were constructed previously (Yang and Rosenwald 2016). High copy plasmids containing a single specific yeast ORF were either isolated from the screened library (*COG4* and *SNX4*) or were obtained from the yeast ORF collection (*SLT2*, *ATG5*, *PEP3*, *IVY1*, and *TAX4*) (Gelperin *et al.* 2005). The pRS316-GFP-Atg8 plasmid was a gift from Daniel Klionsky (University of Michigan) (Suzuki *et al.* 2001). The plasmids used in this study are listed in Supplemental Material, Table S1.

Antibodies used included a mouse anti-GFP (green fluorescent protein) primary antibody (Roche Diagnostics, 11814460001); a mouse anti-phosphoglycerate kinase-1 (Pgk-1) antibody (Molecular Probes, A6457); and a sheep anti-mouse IgG horseradish peroxidase-linked secondary antibody (GE Healthcare, NA931). The enhanced chemiluminescence (ECL) prime kit was from GE Healthcare (RPN2236).

All chemical reagents were from Sigma-Aldrich, unless otherwise noted.

### Genetic screen for high copy number suppressors

The YEp13-based yeast high copy number genomic library (ATCC 37323) (Nasmyth and Reed 1980) was used in the screen. Yeast strains *arl1*Δ and *ypt6*Δ were transformed with the library by using lithium acetate transformation, as described previously (Gietz and Woods 2002), and were plated onto either SC with glucose without leucine medium to determine number of total transformants (20,000 colonies for each strains) or SC with glucose without leucine containing 5 ng/ml rapamycin (Sigma-Aldrich, R0395). We previously determined using this concentration of rapamycin that the negative control, the *atg1*Δ strain, cannot grow at either 30 or 37°, while the wild-type (WT) parent can grow in the presence of rapamycin at both temperatures (Yang and Rosenwald 2016). Here, the rapamycin plates were incubated at 37°. Plasmids from the colonies showing resistance to rapamycin at 37° were isolated (Hoffman and Winston 1987) and transformed into *Escherichia coli* DH5α strain by electroporation, followed by purification from *E. coli* cells (QIAGEN QIAprep Spin Miniprep Kit). Plasmids were then retransformed into *arl1*Δ or *ypt6*Δ strains to confirm that the suppressing function was contained on the plasmid and not as a result of a chromosomal mutation in the original yeast transformant. The plasmids were sequenced with primers (MP10: CTTGGAGCCACTATCGAC, MP11: CCGCACCTGTGGCGCCG) adjacent to the unique *Bam*HI site of YEp13, into which the genomic fragments were cloned. Sequencing was performed by Genewiz (Plainfield, NJ). The sequencing results were analyzed with the Basic Local Alignment Search Tool (BLAST, NCBI) (Altschul *et al.* 1990). The genomic regions contained in the plasmids were identified with the genome browser tool from the *Saccharomyces* genome database (SGD; yeastgenome.org). ORF functions as membrane traffic or autophagy regulators were selected and the high copy number plasmids containing a single ORF of interest were either obtained directly from this screen (some of the plasmids that passed the screen contained only a single gene) or from the yeast ORF collection (GE Dharmacon) (Gelperin *et al.* 2005). These plasmids were transformed into *arl1*Δ and *ypt6*Δ strains to test in more detail whether they suppressed the autophagy defect of *arl1*Δ and *ypt6*Δ strains at 37°. When the ORF collection plasmids were used, cells were grown in media containing galactose rather than glucose because the genes of interest are under the control of the *GAL1* promoter.

### Yeast culture conditions and the induction of autophagy

Yeast strains were cultured in synthetic dropout media (2% glucose and 0.67% yeast nitrogen base without amino acids or uracil, supplemented with appropriate nutrients) (Green and Moehle 2001). For yeast strains containing plasmids from the yeast ORF collection, galactose was used as the carbon source in place of glucose because the genes are under the control of the *GAL1* promoter. To induce autophagy, nitrogen starvation medium (SD-N or SGal-N; 2% glucose or 2% galactose, 0.17% yeast nitrogen base without amino acids, ammonium sulfate, or vitamins) was used. Yeast cells were first cultured in synthetic dropout media lacking appropriate amino acids or uracil depending on the plasmids they contained, until OD_600_ = 0.6. They were then incubated at either 30 or 37° in nonstarvation conditions for 30 min before being washing twice in SD-N (or SGal-N) medium and further incubated in SD-N (or SGal-N) at the same temperature for 3 hr. All chemicals for media were from Fisher Scientific except yeast nitrogen base (Sunrise, 1500-500).

### GFP-Atg8 processing assay

Yeast strains containing the pRS316-GFP-Atg8 plasmid were cultured in starvation medium at 30 or 37° to induce autophagy. Cells were collected and subjected to trichloroacetic acid precipitation, used to extract proteins from cells as described previously (Yang and Rosenwald 2016). Total proteins from 0.8 OD_600_ were separated on precast polyacrylamide gels (Any kD, Bio-Rad, 456–9036). The proteins were transferred onto nitrocellulose membranes and the membrane was incubated with a mouse anti-GFP antibody, followed by an HRP-conjugated anti-mouse secondary antibody. HRP signals were visualized using an ECL prime kit (GE Healthcare) and detected with an ImageQuant LAS 4010 imager (GE Healthcare). Each set of western blot experiments was repeated three times. Representative examples are shown in each figure.

### GFP-Atg8 fluorescence microscopy

Yeast strains containing the pRS316-GFP-Atg8 plasmid were collected from rich medium (SD medium lacking the appropriate nutrients or SGal medium for yeast strains containing the yeast ORF collection plasmids) or starvation medium at 30 or 37°, and washed once with water before imaging. Cells were visualized with a Zeiss AxioImager M2 florescence microscopy system using a 63 × oil lens. Images were captured and deconvolved using Volocity 6.3 (PerkinElmer) software. The fluorescence microscopy experiments were repeated three times.

### Pho8Δ60 assay

The Pho8Δ60 assay to quantify the magnitude of autophagy was performed as previously described (Noda and Klionsky 2008). Briefly, cell lysates from 0.5 OD_600_ of cells were incubated with 5.5 mM α-naphthyl phosphate (disodium salt, Sigma-Aldrich, N7255) for 20 min at 30° in reaction buffer (250 mM Tris-HCl, pH 9.0, 10 mM MgSO_4_, and 10 µM ZnSO_4_). The reaction was stopped with an equal volume of 2 M glycine-NaOH, pH 11.0. Fluorescence emissions of the product 1-napthol were measured (λ_ex_ = 330 and λ_em_ = 472) using a GloMax plate reader (Promega) with a UV filter. Protein concentrations were determined by the Bradford assay (Bradford 1976). All experiments were repeated three times.

### Data availability

The authors state that all data necessary for confirming the conclusions presented in the article are represented fully within the article.

## Results

### Identification of genomic regions that suppress rapamycin sensitivity of arl1Δ and ypt6Δ strains at 37°

Previously, we found that the deletion of either the *ARL1* or *YPT6* gene causes sensitivity to the drug rapamycin only at 37°, and not at 30° (the *atg1*Δ strain, the negative control for autophagy, showed growth defects on rapamycin at both 30 and 37°, while the WT strain was able to grow at both temperatures). We further confirmed that this high temperature growth defect on rapamycin was due to a failure to process autophagy normally (Yang and Rosenwald 2016). To determine what genes might suppress the autophagy defect in these strains, we utilized the rapamycin sensitivity phenotype at 37° as a preliminary way to isolate genomic fragments that contain potential high copy number suppressors. Each deletion strain was transformed with the library and 20,000 colonies each were screened for their ability to grow on rapamycin medium at 37°. As a result, 27 distinct genomic fragments were identified from the screen on the *arl1*Δ strain (Table S2), and 10 were identified for the *ypt6*Δ strain (Table S3).

From the screen results for the *arl1*Δ strain, we selected *COG4*, *TAX4*, *SNX4*, and *SLT2* as candidates for further testing based on their cellular functions as important regulators of membrane traffic or autophagy (see Table S2). We obtained *ARL1* from the screen, a good internal control. Also, *YPT6* was identified from the screen, which was previously confirmed as a suppressor of the *arl1*Δ strain (Yang and Rosenwald 2016). For genes of interest contained on library plasmids with several genes, we purchased the relevant single genes from the ORF collection (Gelperin *et al.* 2005). For the *COG4* and *SNX4* plasmids obtained from the screen, each of these plasmids contained only the gene of interest, thus we used these two plasmids directly in additional assays. For the screen results of the *ypt6*Δ strain, we selected *IVY1*, *ATG5*, and *PEP3* (see Table S3). We also obtained a fragment with *YPT6*, again a good internal control. In summary, we selected a total of seven genes to be tested specifically for suppression of the autophagy defects. All seven plasmids containing single gene candidates were transformed into the *arl1*Δ and *ypt6*Δ strains.

### Identification of high copy suppressors of the arl1Δ strain

**Figure 1:**
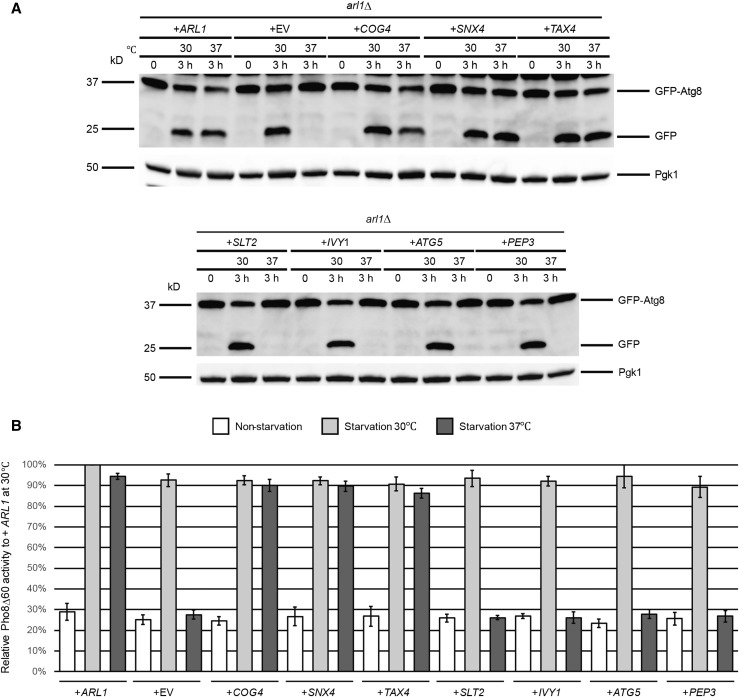
Autophagy-specific assays show *COG4*, *TAX4*, and *SNX4* suppress the temperature-sensitive autophagy defect of the *arl1*Δ strain. (A) GFP-Atg8 degradation was increased at 37° in the *arl1*Δ strain when transformed with plasmids containing *COG4*, *TAX4*, and *SNX4*, as well as WT *ARL1*. All strains were cultured at 30° in the appropriate nonstarvation medium (containing either glucose or galactose) until log-phase, then all the strains were incubated at 37 or 30° for 30 min. The cells were then washed twice with SD-N (or SGal-N) medium, and cultured in SD-N (or SGal-N) for 3 hr either at 37 or at 30°. (B) *COG4*, *TAX4*, and *SNX4* increased the Pho8Δ60 activities in *arl1*Δ (YSA003) at 37°. Error bars represent SD from three biological replicates. GFP, green fluorescent protein; SD-N, glucose nitrogen starvation medium; SGal-N, galactose nitrogen starvation medium; WT, wild-type.

We first transformed high copy plasmids containing *COG4*, *SNX4*, *SLT2*, *TAX4*, *IVY1*, *ATG5*, and *PEP3*, as well as *ARL1* and empty vector (EV, YEp352) into the *arl1*Δ strain. In plasmids containing *SLT2*, *TAX4*, *IVY1*, *ATG5*, and *PEP3*, expression is controlled by the *GAL1* promoter, thus, yeast with these plasmids were cultured in medium with galactose as the carbon source. The *COG4*- and *SNX4*-containing plasmids were isolated from the YEp13 library as fragments with a single gene and expression of these genes is under the control of their native promoters; thus, yeast containing these two plasmids were cultured using normal growth conditions with glucose as the carbon source. In order to test which of the seven candidates suppressed the autophagy defect at 37°, an assay that followed cleavage of a modified version of the protein Atg8, GFP-Atg8, was used to monitor the transport of Atg8 to the vacuole (Figure 1A). Once autophagy is triggered, Atg4 modifies Atg8 on its C-terminus. This modification enables Atg8 to be conjugated to PE on its C-terminus, which helps expand the membranes of the phagophore to become the autophagosome (Xie *et al.* 2008). When the autophagosome fuses with the vacuole, Atg8 is degraded. However, when the fusion protein GFP-Atg8 is transferred to the vacuole, Atg8 is degraded, but the GFP moiety is resistant to degradation and can therefore be detected as free GFP on western blots if autophagy proceeds normally. As the results show, at time 0, no free GFP was detected in any of the strains. When the cells were treated in starvation medium for 3 hr at 30°, free GFP could be detected in all strains, demonstrating normal autophagy. As the negative control, no free GFP could be detected in the cell with empty vector at 37°, meaning that Arl1 is required for autophagy at high temperature, as previously found (Yang and Rosenwald 2016). Cells overexpressing *COG4*, *TAX4*, *SNX4*, and *ARL1* were able to process GFP-Atg8 normally at 37°, while overexpression of *IVY1*, *ATG5*, *SLT2*, or *PEP3* did not suppress the autophagy defect of the *arl1*Δ strain at 37°.

In *S. cerevisiae*, the activity of a N-terminally truncated form of the vacuolar alkaline phosphatase Pho8 (Pho8Δ60) is widely used to measure the magnitude of autophagy (Noda and Klionsky 2008). Normally, Pho8 is transported to the vacuole via the secretory pathway, where it is processed and activated to form the mature form by proteolysis. Upon removal of the *N*-terminal 60 amino acids, Pho8 can only be transported to the vacuole by autophagy. Thus, the amount of Pho8 enzymatic activity under these conditions is a measure of autophagy. We performed the Pho8Δ60 assay in the *arl1*Δ (YSA003) strain, containing the *PHO8*Δ*60* allele after transformation with plasmids bearing the eight genes of interest (Figure 1B). *ARL1* and empty vector (YEp352) were used as controls. The results show that, consistent with the GFP-Atg8 assay, overexpression of *COG4*, *TAX4*, *SNX4*, and *ARL1* was able to suppress the autophagy defect of the *arl1*Δ strain at 37°, whereas overexpression of *IVY1*, *ATG5*, *SLT2*, and *PEP3* was not.

**Figure 2:**
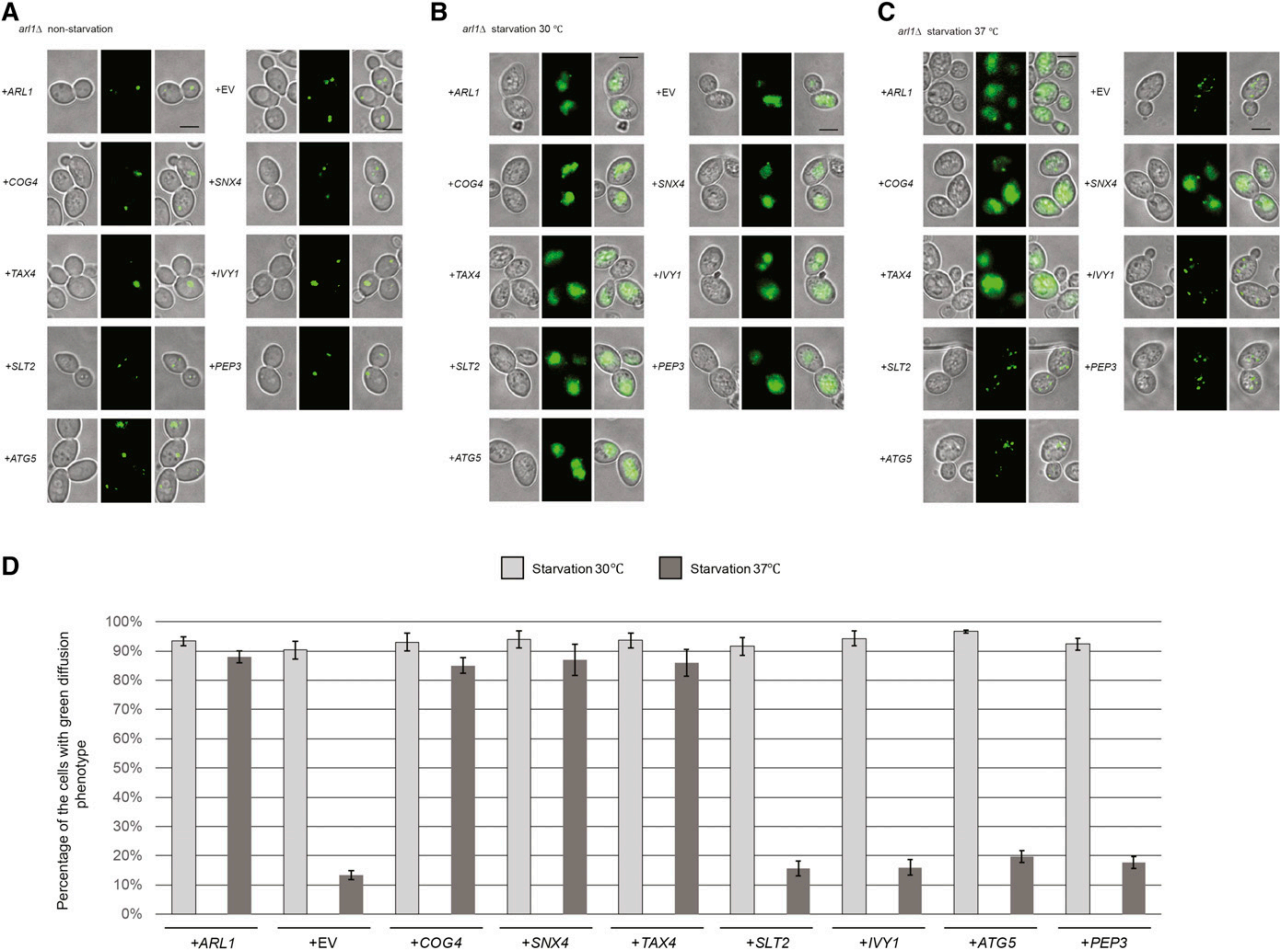
*COG4*, *TAX4*, and *SNX4* suppress the GFP-Atg8 processing defect at 37° in the *arl1*Δ strain. Cells were grown then starved for nitrogen as described. (A) Fluorescence images of all strains in nonstarvation conditions. (B) Fluorescence images of all strains in starvation conditions at 30°. (C) Fluorescence images of all strains in starvation conditions at 37°. (D) The percentage of the cells with the green diffusion phenotype in starvation conditions at 30 and 37°. At least 100 cells were counted for each strain. Error bars represent SD from three biological replicates. Scale bar: 3 μm. EV, empty vector.

We also visualized the transport of GFP-Atg8 by fluorescence microscopy (Figure 2). Under nonstarvation conditions, GFP-Atg8 aggregated as single dots in the cells, denoting the PAS (Suzuki *et al.* 2001) (Figure 2A). When cells were treated with starvation medium at 30°, all of the strains had a vacuolar diffuse green phenotype (Yang and Rosenwald 2016), indicating the normal processing of GFP-Atg8. Under starvation conditions at 37° (Figure 2C), cells overexpressing *COG4*, *SNX4*, and *TAX4* as well as *ARL1* had the green diffuse phenotype, consistent with the conclusion from Figure 1 that these four genes, when overexpressed, are able to suppress the autophagy defect in the *arl1*Δ strain. On the other hand, cells overexpressing *IVY1*, *PEP3*, *SLT2*, and *ATG5* exhibited multiple green dots, suggesting defective autophagy (Figure 2C). After counting the cells with diffuse green phenotype (normal autophagy) at both 30 and 37°, we found that the *arl1*Δ strains that overexpressed *COG4*, *SNX4*, and *TAX4* had a significantly higher percentage of cells with the diffuse green phenotype at 37°, while cells overexpressing *IVY1*, *PEP3*, *SLT2*, and *ATG5* had a dramatically decreased percentage of cells with this phenotype at high temperature compared with 30°. In conclusion, we identified *COG4*, *SNX4*, and *TAX4* as high copy suppressors for the high temperature autophagy defect of the *arl1*Δ strain.

### Identification of high copy suppressors of the ypt6Δ strain

**Figure 3:**
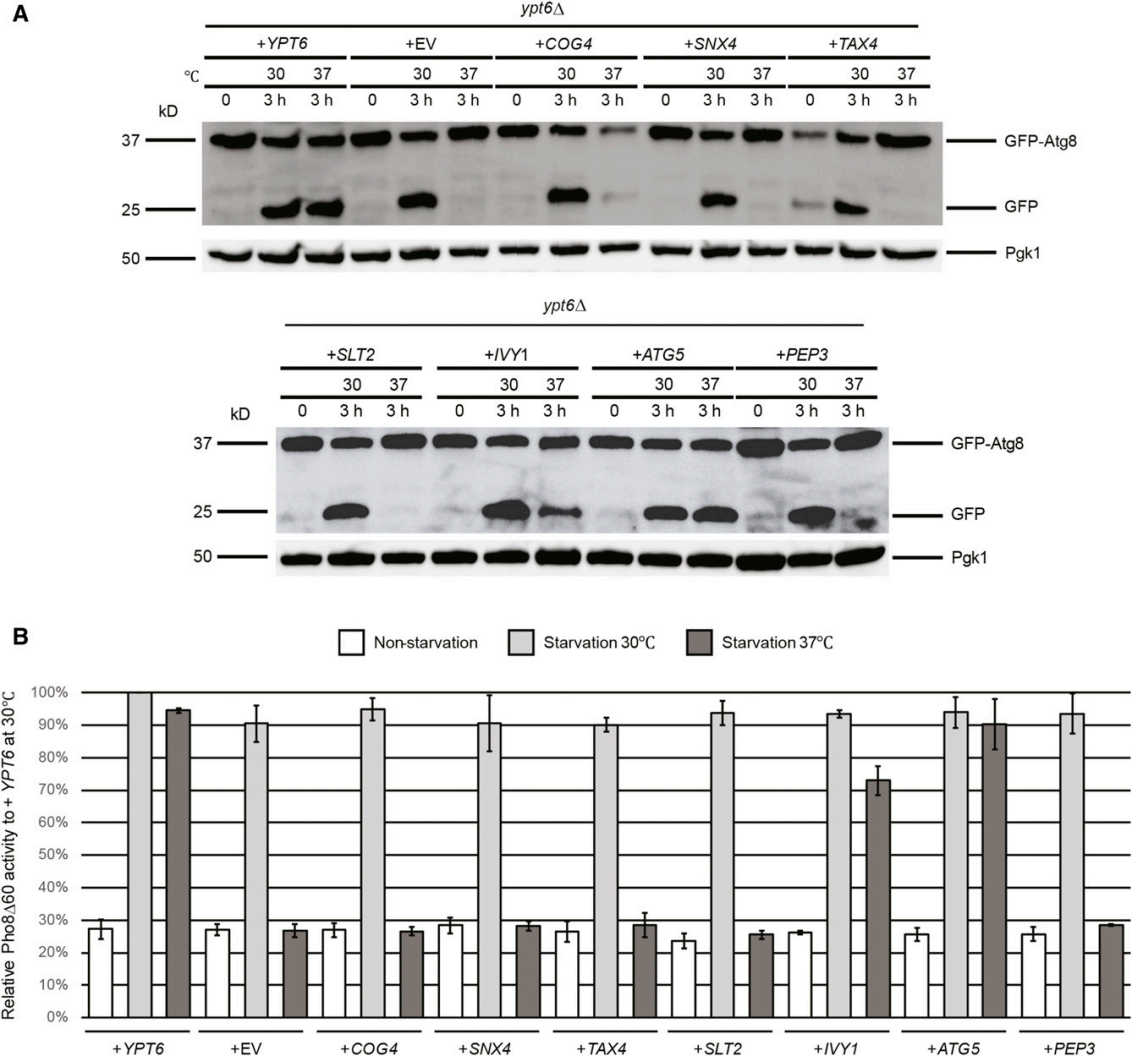
Autophagy-specific assays show *IVY1* and *ATG5* suppress the temperature-sensitive autophagy defect of the *ypt6*Δ strain. (A) GFP-Atg8 degradation was increased at 37° in *ypt6*Δ when transformed with plasmids containing *IVY1*, *ATG5*, and WT *YPT6*. (B) *IVY1* and *ATG5* increased the Pho8Δ60 activities in *ypt6*Δ (YSA004) at 37°. Error bars represent SD from three biological replicates. Methods are the same as in Figure 1. GFP, green fluorescent protein; WT, wild-type.

**Figure 4:**
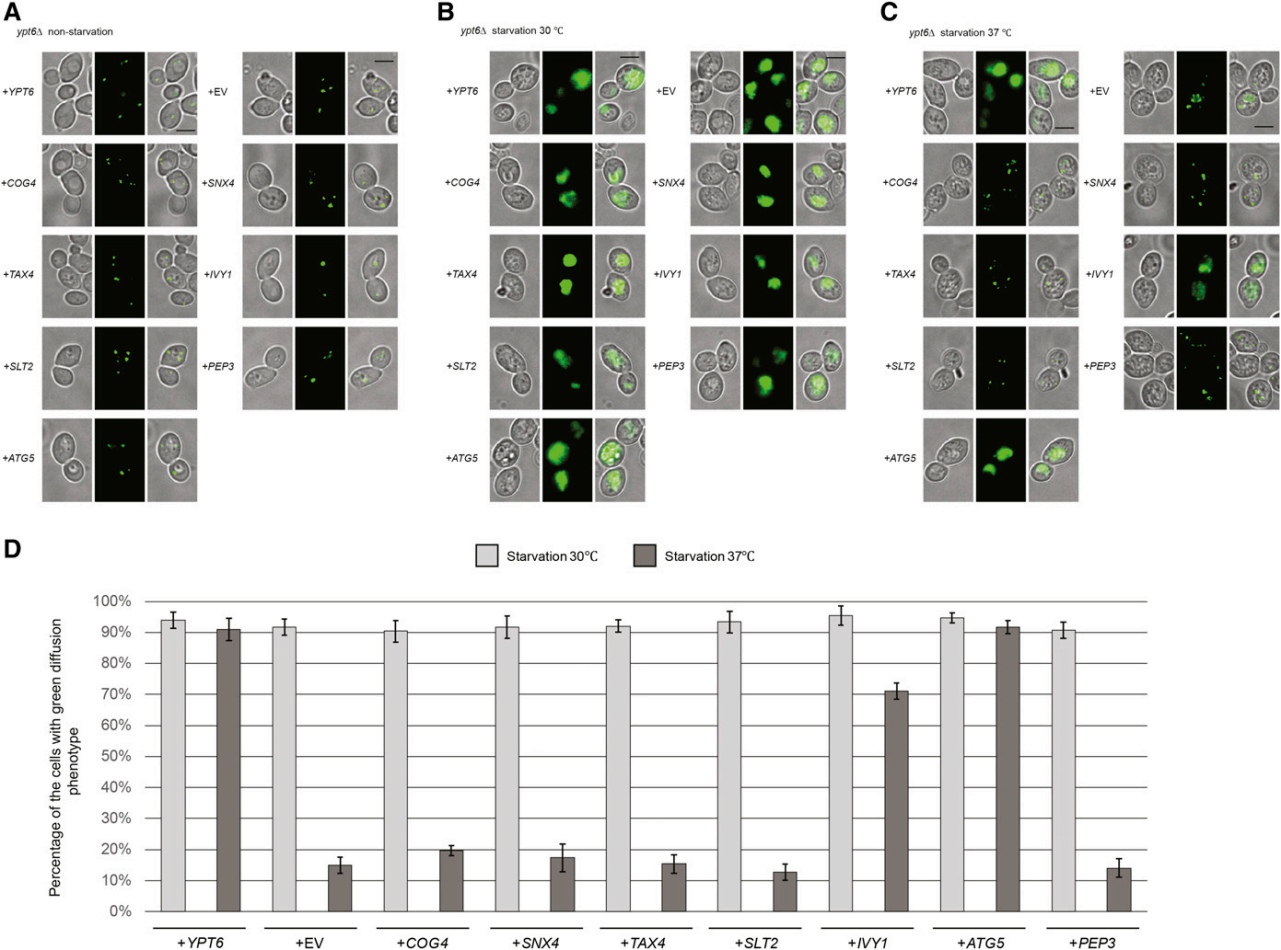
*IVY1* and *ATG5* suppress the GFP-Atg8 processing defect at 37° in the *ypt6*Δ strain. Cells were grown then starved for nitrogen as described. (A) Fluorescence images of all strains in nonstarvation conditions. (B) Fluorescence images of all strains in starvation conditions at 30°. (C) Fluorescence images of all strains in starvation conditions at 37°. (D) The percentage of the cells with the green diffusion phenotypes in starvation condition at 30 and 37°. At least 100 cells were counted for each strain. Error bars represent SD from three biological replicates. Scale bar: 3 μm. EV, empty vector.

We performed the same set of autophagy assays as for the *arl1*Δ strain to test which of the seven genes from the screen were able to suppress the high temperature autophagy defect. From the GFP-Atg8 assay, we found that only *IVY1* and *ATG5*, as well as the positive control *YPT6*, were able to suppress the autophagy defect of the *ypt6*Δ strain at 37°, as demonstrated by the appearance of free GFP (Figure 3A). This conclusion was further confirmed through Pho8Δ60 assay (Figure 3B); upon overexpression of *IVY1*, *ATG5*, or *YPT6*, the Pho8Δ60 activity in the *ypt6*Δ strain (YSA004) was increased compared with empty vector. Similarly, these results were confirmed by the GFP-Atg8 fluorescence phenotype (Figure 4, A–C), as we found that the *ypt6*Δ strain overexpressing *IVY1* or *ATG5* had an increased percentage of cells with the diffuse green phenotype at 37° (Figure 4D). In conclusion, we identified *IVY1* and *ATG5* as high copy suppressors for the high temperature autophagy defect of the *ypt6*Δ strain.

**Figure 5:**
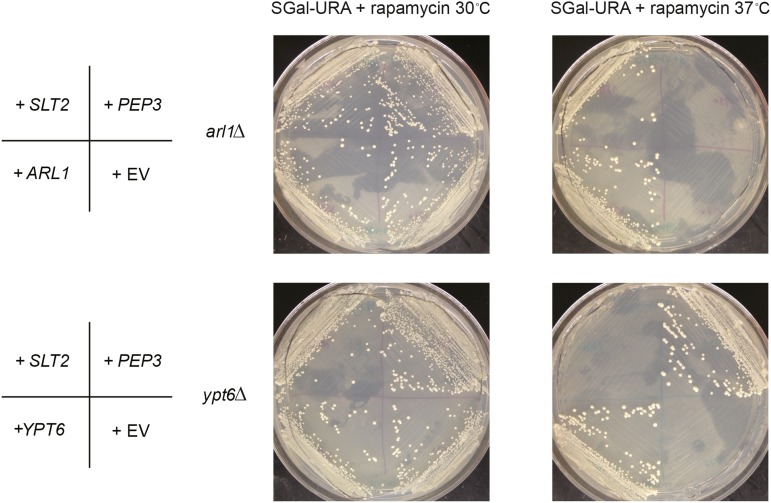
*SLT2* and *PEP3* suppress the rapamycin sensitivity at 37° of the *arl1*Δ or *ypt6*Δ strains, respectively. SGal-URA (galactose‐ containing medium without uracil) containing 5 ng/ml rapamycin was used in this assay. The images are taken on day 10 of growth.

From the seven candidates, we found that *COG4*, *TAX4*, and *SNX4* suppressed the autophagy defect of the *arl1*Δ strain, while *IVY1* and *ATG5* suppressed the defect in the *ypt6*Δ strain. Two genes identified in the primary screen, *SLT2* and *PEP3*, selected from rapamycin sensitivity screens for the *arl1*Δ and *ypt6*Δ strains, respectively, did not suppress the defect in either strain as measured by the more specific assays. In order to confirm that these two genes were actually the relevant ones in the genomic fragments, we streaked the *arl1*Δ strain with a plasmid containing only the *SLT2* gene and the *ypt6*Δ strain with a plasmid containing only the *PEP3* gene onto medium containing 5 ng/ml rapamycin, and cultured them at 37°. These genes, *SLT2* and *PEP3*, suppressed the high temperature rapamycin sensitivity of *arl1*Δ and *ypt6*Δ, respectively (Figure 5). These results indicate that genes that suppressed the rapamycin phenotype in these strains are not necessarily regulators of autophagy.

## Discussion

Autophagy is an essential cellular process that helps cells overcome stressful environmental conditions. It is also tightly regulated and requires a large number of proteins to perform correctly. The cooperation and interactions between different regulators are particularly important for the correct execution of autophagy. Autophagy relies intensively on membrane resources for the formation of the autophagosome to sequester the targeted cytosolic components. Therefore, many membrane traffic regulators, including many monomeric GTP-binding proteins, have been shown to be essential for this process. Examples include Ypt1 and Sec4. While Ypt1 normally controls the traffic between the ER and the *cis*-Golgi (Jedd *et al.* 1995) and Sec4 is required for the exocytic secretion pathway (Kabcenell *et al.* 1990), both are required for membrane transport to the PAS to form the autophagosome (Geng *et al.* 2010) (Lynch-Day *et al.* 2010).

Previously, we discovered that Arl1 and Ypt6, the two small GTP-binding proteins that regulate late Golgi vesicle traffic, also have roles in autophagy (Yang and Rosenwald 2016). Interestingly, we discovered that a simple knockout of one of the two genes did not impair autophagy at 30°, yet both *arl1*Δ and *ypt6*Δ strains showed completely defective autophagy at 37°. Furthermore, we found that Arl1 and Ypt6 participate in autophagy through interactions with the GARP complex. Retention of both Arl1 and Ypt6 is required for the proper localization of the GARP complex to the PAS at 37°, and thus affects membrane transport from the TGN to the PAS for autophagosome formation. An absence of one of the two is apparently no longer sufficient to securely bind GARP to the PAS at high temperature. Because the autophagy pathway is very complicated, we sought to identify other regulators that work cooperatively with Arl1 or Ypt6 to affect autophagy under high temperature stress.

Many widely used autophagy-specific assays are challenging to adapt for high copy suppressor screening, due to the difficulty in manipulating large sample numbers for western blot analysis (GFP-Atg8 proteolysis), enzymatic activity (Pho8Δ60), or microscopy (GFP-Atg8 localization). Thus, we chose to use the rapamycin sensitivity assay as a preliminary screen, since we found that this phenotype correlated with phenotypes of the more specific assays. Rapamycin blocks the kinase function of TORC1, and in turn activates many TORC1-controlled pathways, including autophagy. However, because TORC1 regulates many pathways, such as the ribosome biogenesis pathway (Martin *et al.* 2004), cell cycle (Barbet *et al.* 1996), amino acid biosynthesis pathways (Komeili *et al.* 2000; Crespo *et al.* 2002), and the cell wall integrity pathway (Torres *et al.* 2002), the suppressors identified from this screen may not specifically suppress the autophagy defect of the *arl1*Δ and *ypt6*Δ strains. Therefore, we selected genes that express regulators for membrane traffic or autophagy, since the expectation would be that such genes could potentially be specific regulators of autophagy. Using this approach, we found that *COG4*, *TAX4*, and *SNX4* suppressed the autophagy defect of the *arl1*Δ strain, while *IVY1* and *ATG5* suppressed the defect of the *ypt6*Δ strain.

Cog4 is one of the subunits of the conserved oligomeric Golgi (COG) complex, a tethering complex that is similar to GARP. The COG complex contains eight subunits (Cog1–Cog8), and its structure can be divided into two lobes: lobe A consists of Cog2, Cog3, and Cog4 and lobe B consists of Cog5, Cog6, Cog7, and Cog8, while Cog1 connects the two lobes (VanRheenen *et al.* 1999; Kim *et al.* 2001; Ram *et al.* 2002). The COG complex mediates vesicle traffic within the Golgi apparatus, between the Golgi and endosomes, as well as between the ER and Golgi (VanRheenen *et al.* 1999; Suvorova *et al.* 2002; Bruinsma *et al.* 2004; Oka *et al.* 2004; Zolov and Lupashin 2005). In addition, lobe A is directly involved in autophagy because it is localized to the PAS and required for the formation of the autophagosome (Yen *et al.* 2010).

Tax4 regulates phosphatidylinositol 4,5-bisphosphate levels in the cell by activating the PtdIns phosphatase Inp51p (Morales-Johansson *et al.* 2004). It localizes to the PAS upon triggering of autophagy and mediates the recruitment of Atg17 to the PAS, thus affecting the early stages of autophagy. Interestingly, Tax4 is required for the normal expression of *VPS51*, one of the subunits of the GARP complex (Bugnicourt *et al.* 2008), suggesting an interaction between Tax4 and the GARP complex specifically in autophagy. We hypothesize that the overexpression of *COG4* or *TAX4* can either provide more membrane to the PAS to form the autophagosome, or recruit more regulators such as Atg17 to increase the extent of autophagy, in the *arl1*Δ strain background.

Snx4 is a sorting nexin that controls the recycling of some late-Golgi SNAREs (*i.e.*, Snc1) between the early endosome and the TGN (Hettema *et al.* 2003). Previous results suggested that Snx4 is required for a selective form of autophagy, the cytoplasm-to-vacuole transport pathway (Nice *et al.* 2002), although our results suggest it may also have a role in macroautophagy as well.

Atg5 itself is an essential autophagy regulator, and functions to mediate the lipidation of Atg8, therefore increasing the elongation of the phagophore for autophagosome formation (Romanov *et al.* 2012; Sakoh-Nakatogawa *et al.* 2013). We hypothesize that in the *ypt6*Δ strain, the overexpression of *ATG5* suppresses the autophagy defect by increasing the formation of autophagosomes.

Previous results have shown that Ivy1 localizes to the vacuole and is an effector of the small GTP-binding protein Ypt7. Ivy1 controls the function of TORC1 in combination with the V-ATPase, as the strain with a double deletion of *IVY1* and one of the subunits of the V-ATPase *VAM16* cannot grow in the presence of rapamycin (Numrich *et al.* 2015). This double mutant strain has an invaginated vacuolar surface phenotype, which suggests that Ivy1, together with the V-ATPase, functions to maintain vacuolar shape as well as regulate the function of TORC1 (Numrich *et al.* 2015). Ivy1 may function by increasing the fusion between the autophagosome and the vacuole as it is required for the integrity of the vacuole membrane. We observed that *IVY1* is a weaker suppressor of the autophagy activity of *ypt6*Δ compared to *ATG5* (see the Pho8Δ60 assay in Figure 3B). This result may due to the fact that Ivy1 is indirectly involved in autophagosome formation.

Besides the five genes we confirmed to be the suppressors of the autophagy defect of the *arl1*Δ or *ypt6*Δ strains, we also selected *SLT2* and *PEP3* for study. Slt2 is a MAP kinase that regulates the protein kinase C pathway (Madden *et al.* 1997) and mediates two selective forms of autophagy, mitophagy to clear the damaged mitochondria (Mao *et al.* 2011) and pexophagy for peroxisome clearance. Pep3 is a subunit of the CORVET (class C core vacuole/endosome tethering membrane) tethering complex and is required for the biogenesis of the vacuole (Preston *et al.* 1991; Srivastava *et al.* 2000). Although *SLT2* and *PEP3* did not suppress the autophagy defect of the *arl1*Δ or *ypt6*Δ strain, respectively, they did suppress the growth defect of the appropriate strain on rapamycin plates at 37°.

We conducted the two rapamycin screens concurrently, with the expectation that genes identified in one strain background might suppress in the other background. This expectation was based on our previous finding that *YPT6* suppressed the autophagy defect of the *arl1*Δ strain (Yang and Rosenwald 2016). We expected that *IVY1* and *ATG5* would also suppress the *arl1*Δ strain’s autophagy defect because, based on our results, we hypothesized that Ivy1 and Atg5 function downstream of Ypt6. However, we found that, other than *YPT6* itself, none of the genes identified as suppressors of one strain suppressed the other strain. In other words, *COG4*, *TAX4*, and *SNX4* selected from the screen of the *arl1*Δ strain only suppressed the autophagy defect in the *arl1*Δ strain, not the *ypt6*Δ strain, whereas *IVY1* and *ATG5* only suppressed the defect in the *ypt6*Δ strain, not the *arl1*Δ strain. Previously, we showed Arl1 and Ypt6 work together to mediate autophagy, as the conditional knockout of both the *ARL1* and *YPT6* genes can lead to a complete block of autophagy, even at 30°. The fact that they do not share the same set of high copy suppressors may suggest that they have independent roles in some aspects of autophagy as well. Moreover, the fact that *SLT2* and *PEP3* are rapamycin sensitivity suppressors but cannot suppress the autophagy defects in the *arl1*Δ or *ypt6*Δ strain may suggest that Arl1 and Ypt6 regulate TORC1 function; however, at this point, we cannot rule out the possibility that *SLT2* and *PEP3* are bypass suppressors of the loss of *ARL1* and *YPT6*, respectively.

In conclusion, we identified several genes that suppress the high temperature autophagy defect in cells lacking either *ARL1* or *YPT6*. Although researchers have uncovered a great deal about the molecular mechanisms responsible for autophagy, additional work will be necessary to uncover the detailed relationships between the suppressors we uncovered in this study and these two monomeric G proteins. From this analysis, we hope to learn more about the signaling pathways necessary for a comprehensive understanding of autophagy.

## Supplementary Material

Supplemental material is available online at www.g3journal.org/lookup/suppl/doi:10.1534/g3.116.035998/-/DC1.
