# Supplementary material for: A High Copy Suppressor Screen for Autophagy Defects in Saccharomyces arl1Δ and ypt6Δ Strains
Source: G3 (Bethesda). 2016 Dec 12;7(2):333–41. doi: 10.1534/g3.116.035998 (PMC5295583; doi:10.1534/g3.116.035998)
Supplement: Supplementary file 1 [file 333FileS1.pdf]

REFERENCES

Gelperin, D. M., M. A. White, M. L. Wilkinson, Y. Kon, L. A. Kung *et al.*, 2005 Biochemical and genetic analysis of the yeast proteome with a movable ORF collection. Genes Dev 19**:** 2816-2826.

Hill, J. E., A. M. Myers, T. J. Koerner and A. Tzagoloff, 1986 Yeast/E. coli shuttle vectors with multiple unique restriction sites. Yeast 2**:** 163-167.

Nasmyth, K. A., and S. I. Reed, 1980 Isolation of genes by complementation in yeast: molecular cloning of a cell-cycle gene. Proc Natl Acad Sci U S A 77**:** 2119-2123.

Rosenwald, A. G., M. A. Rhodes, H. Van Valkenburgh, V. Palanivel, G. Chapman *et al.*, 2002 ARL1 and membrane traffic in Saccharomyces cerevisiae. Yeast 19**:** 1039-1056.

Suzuki, K., T. Kirisako, Y. Kamada, N. Mizushima, T. Noda *et al.*, 2001 The pre-autophagosomal structure organized by concerted functions of APG genes is essential for autophagosome formation. EMBO J 20**:** 5971-5981.

Yang, S., and A. G. Rosenwald, 2016 Autophagy in Saccharomyces cerevisiae requires the monomeric GTP-binding proteins, Arl1 and Ypt6. Autophagy**:** 1-17.
