# Supplementary material for: A High Copy Suppressor Screen for Autophagy Defects in Saccharomyces arl1Δ and ypt6Δ Strains
Source: G3 (Bethesda). 2016 Dec 12;7(2):333–41. doi: 10.1534/g3.116.035998 (PMC5295583; doi:10.1534/g3.116.035998)
Supplement: Supplementary file 2 [file 333TableS1.pdf]

Table S1 Plasmids used in the study

Plasmid name	Description	Sources
pGFP-AUT7	pRS316-GFP-ATG8	(SUZUKI *et al.* 2001)
YEp352	2μ vector, *URA3*	(HILL *et al.* 1986)
pARY1-3	*ARL1* (wild type) in YEp352	(ROSENWALD *et al.* 2002)
pRS316-YPT6	*YPT6* (wild type) in pRS316	(YANG AND ROSENWALD 2016)
YEp13-COG4	*COG4* in YEp13	(NASMYTH AND REED 1980)
YEp13-SNX4	*SNX4* in YEp13	(NASMYTH AND REED 1980)
pTAX4	*TAX4* in pGB1805	(GELPERIN *et al.* 2005)
pIVY1	*IVY1* in pGB1805	(GELPERIN *et al.* 2005)
pATG5	*ATG5* in pGB1805	(GELPERIN *et al.* 2005)
pPEP3	*PEP3* in pGB1805	(GELPERIN *et al.* 2005)
pSLT2	*SLT2* in pGB1805	(GELPERIN *et al.* 2005)
