# Supplementary material for: A High Copy Suppressor Screen for Autophagy Defects in Saccharomyces arl1Δ and ypt6Δ Strains
Source: G3 (Bethesda). 2016 Dec 12;7(2):333–41. doi: 10.1534/g3.116.035998 (PMC5295583; doi:10.1534/g3.116.035998)
Supplement: Supplementary file 3 [file 333TableS2.pdf]

Table S2. Genomic fragments identified from the high copy suppressors for *arl1*Δ.

**Chromosomal Position**	**Number of isolates**	**ORFs**	**Descriptions of proteins expressed by the potential suppressors genes (membrane traffic and autophagy regulators)**
II 565073..575246	3	*YSY6;* ***ARL1****; UBS1; TYR1; POP7; EXO5; PEX32*	ARF like small GTP binding protein.
XVI 736052..739759	1	***COG4***	Essential component of the COG complex, intra-Golgi retrograde traffic.
XII 668013..669540	1	***YPT6****; VPS63*	Small GTP-binding protein from the Rab family.
XII 667891..670087	1	***YPT6****; VPS63; TMA7*	Small GTP-binding protein from the Rab family.
X 274283..280632	1	*ALY2; TAX4*	EH domain-containing protein; involved in regulating phosphatidylinositol 4,5-bisphosphate levels and autophagy, for targeting Atg17 to PAS.
X 377905..380245	2	***SNX4***	Sorting nexin; involved in retrieval of late-Golgi SNAREs from post-Golgi endosomes to the trans-Golgi network and in cytoplasm to vacuole transport.
VIII 165120..171159	1	*YHI9;* ***SLT2****; DAP2 (partial)*	Serine/threonine MAP kinase, required for mitophagy and pexophagy.
I 164454..168712	1	*BUD14 (Partial)*	
II 153600..157666	1	*STU1 partial*	
II 591904..599696	1	*SMP1; YBR182c-a; YPC1; YBR184w; MBA1*	
II 647822..652951	1	*NGR1; MET8; SDS24*	
IV 453510..463729	1	*RCR2; RAD57; MAF1; SOK1; TRP1*	
IV 660744..666661	1	*SPO71; TMS1*	
IV 1014928..1024019	1	*RNH202; RRP45; PHM6*	
V 518873..524809	1	*BCK2 (partial); CCA1; RPH1 (partial)*	
X 655992..661260	1	*VPS70; RSF2 (Partial)*	

Table S2. (cont.)

Chromosomal Position	Number of isolates	ORFs	Descriptions of proteins expressed by the potential suppressors genes (membrane traffic and autophagy regulators)
XI 26540..34270	1	*DOA1; OXP1; YRA2*	
XII 824347..825355	1	*KAP95 (Partial); DIC1*	
XIII 293842..298645	1	*SEC59*	
XIV 400634..406605	1	*NCS2; DCP2*	
XIV 425995..431126	1	*MET4*	
XIV 438449..444295	1	*RAS2; OCA1; PHO23; RPS7B*	
XIV 484113..486011	2	*APJ1; MKS2*	
XIV 499400..504947	1	*YNL067W-B; RPL9B; SUN4; AQR1 (partial)*	
XIV 736086..743426	1	*MNT4; FRE4; YNR061C*	
XV 869387..871541	1	*YOR296w*	
XVI 764542..770706	1	*MRI1*	
