# Supplementary material for: A High Copy Suppressor Screen for Autophagy Defects in Saccharomyces arl1Δ and ypt6Δ Strains
Source: G3 (Bethesda). 2016 Dec 12;7(2):333–41. doi: 10.1534/g3.116.035998 (PMC5295583; doi:10.1534/g3.116.035998)
Supplement: Supplementary file 4 [file 333TableS3.pdf]

Table S3. Genomic fragments identified from the high copy suppressors for *ypt6*Δ.

Chromosomal Position	Number of isolates	ORFs	Descriptions of proteins expressed by the potential suppressors genes (membrane traffic and autophagy regulators)
XII:666378..674078	1	*VPS63*, ***YPT6***, *TMA7*, *RED1*, *RPS28B*	Rab family GTPase; Ras-like GTP binding protein involved in the secretory pathway, required for fusion of endosome-derived vesicles with the late Golgi.
XVI 266824..272516	1	*YPL150W*, ***ATG5***	Undergoes conjugation with Atg12p to form a complex involved in Atg8p lipidation.
IV 924504..930158	1	***IVY1***, *COX20*, *HEM1*	Phospholipid-binding protein that interacts with both Ypt7p and Vps33p, localizes to the rim of the vacuole as cells approach stationary phase.
XII:434079..453601	1	***PEP3***, *YLR149c*, *PCD1*, *YLR152c*, *ACS2*, *RNH203*	Component of CORVET membrane tethering complex; vacuolar peripheral membrane protein that promotes vesicular docking/fusion reactions in conjunction with SNARE proteins, required for vacuolar biogenesis.
III:8145..14287	1	*VBA3*	
IV:829784..836624	1	*ATC1*, *UPS3*, *YDR186c*	
VII:33417..36181	1	*PDE1*, *ZIP2*	
XII:461554..462885	1	*YLR154C-G*	
XIII:29734..34906	1	*NGL3*, *TML119w*	
XVI:832730..840723	1	*PIN3*, *NCA2*, *TPO3*	
